# Supplementary material for: Intravenous treatment with a molecular chaperone designed against β-amyloid toxicity improves Alzheimer’s disease pathology in mouse models
Source: Mol Ther. 2022 Aug 17;31(2):487–502. doi: 10.1016/j.ymthe.2022.08.010 (PMC9931549; doi:10.1016/j.ymthe.2022.08.010)
Supplement: Document S1. Figure S1 [file mmc1.pdf]

YMTHE, Volume *31*

Supplemental Information

Intravenous treatment with a molecular chaperone designed against β-amyloid toxicity improves Alzheimer's disease pathology in mouse models

Shaffi Manchanda, Lorena Galan-Acosta, Axel Abelein, Simone Tambaro, Gefei Chen, Per Nilsson, and Jan Johansson

Supplementary material to

Intravenous treatment with a molecular chaperone designed against amyloid-β toxicity improves features of Alzheimer disease pathology in mouse models

Shaffi Manchanda[1,2], Lorena Galan-Acosta[1,2], Axel Abelein[1,2], Simone Tambaro[2], Gefei Chen[1,2], Per Nilsson[2] and Jan Johansson[1,2,*]

[1]Department of Biosciences and Nutrition, Karolinska Institutet, Neo, 141 83 Huddinge, Sweden

[2]Department of Neurobiology, Care Sciences and Society, Division of Neurogeriatrics, Karolinska Institutet, 171 64 Stockholm, Sweden

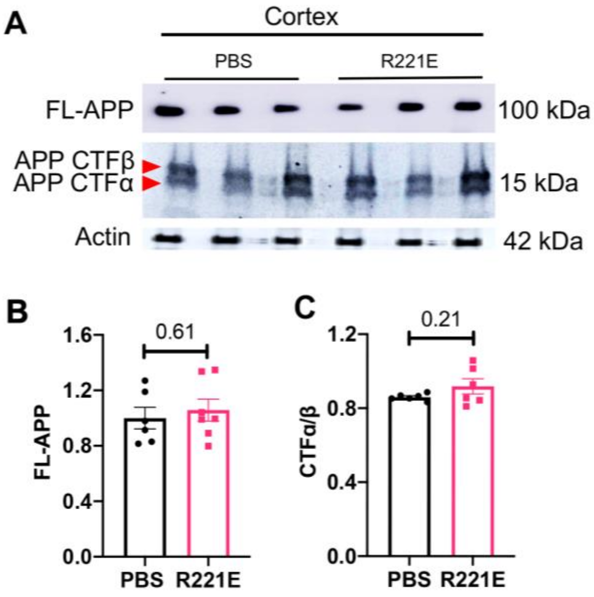

Supplementary Fig. S1. Full-length APP and its processed forms levels remain unchanged after Rh Bri2 BRICHOS R221E treatment in *App*$^{NL\text{-}F}$ mice.

Representative western blots (A) and histograms (B, C) showing levels of full-length APP (FL-APP) and ratio of APP derived C-terminal fragments (CTFα and CTFβ) in cortex of PBS and rh Bri2 BRICHOS R221E treated *App*$^{NL\text{-}F}$ mice (n=6-7 mice/group). Data are represented as Mean ± *SEM*. Unpaired parametric two-tailed t-test was used to calculate the p-values.
