# Supplementary material for: Intravenous treatment with a molecular chaperone designed against β-amyloid toxicity improves Alzheimer’s disease pathology in mouse models
Source: Mol Ther. 2022 Aug 17;31(2):487–502. doi: 10.1016/j.ymthe.2022.08.010 (PMC9931549; doi:10.1016/j.ymthe.2022.08.010)
Supplement: Document S2. Article plus supplemental information [file mmc2.pdf]

Molecular Therapy
Original Article

Intravenous treatment with a molecular chaperone designed against β-amyloid toxicity improves Alzheimer's disease pathology in mouse models

Shaffi Manchanda,[1,2] Lorena Galan-Acosta,[1,2] Axel Abelein,[1,2] Simone Tambaro,[2] Gefei Chen,[1,2] Per Nilsson,[2] and Jan Johansson[1,2]

[1]Department of Biosciences and Nutrition, Karolinska Institutet, Neo, 141 83 Huddinge, Sweden; [2]Department of Neurobiology, Care Sciences and Society, Division of Neurogeriatrics, Karolinska Institutet, 171 64 Stockholm, Sweden

Attempts to treat Alzheimer's disease with immunotherapy against the β-amyloid (Aβ) peptide or with enzyme inhibitors to reduce Aβ production have not yet resulted in effective treatment, suggesting that alternative strategies may be useful. Here we explore the possibility of targeting the toxicity associated with Aβ aggregation by using the recombinant human (rh) Bri2 BRICHOS chaperone domain, mutated to act selectively against Aβ42 oligomer generation and neurotoxicity *in vitro*. We find that treatment of Aβ precursor protein (*App*) knockin mice with repeated intravenous injections of rh Bri2 BRICHOS R221E, from an age close to the start of development of Alzheimer's disease-like pathology, improves recognition and working memory, as assessed using novel object recognition and Y maze tests, and reduces Aβ plaque deposition and activation of astrocytes and microglia. When treatment was started about 4 months after Alzheimer's disease-like pathology was already established, memory improvement was not detected, but Aβ plaque deposition and gliosis were reduced, and substantially reduced astrocyte accumulation in the vicinity of Aβ plaques was observed. The degrees of treatment effects observed in the *App* knockin mouse models apparently correlate with the amounts of Bri2 BRICHOS detected in brain sections after the end of the treatment period.

INTRODUCTION

Age-associated decline of protective mechanisms leads to disruption of protein homeostasis and accumulation of incorrectly folded polypeptides, which can be toxic to cells.[1] To date, 36 proteins have been linked to human amyloid diseases,[2] and Alzheimer's disease (AD) is one of these, where β-amyloid precursor protein (APP) releases amyloidogenic β-amyloid (Aβ) peptides after proteolytic processing.[3] Histopathological characteristics of AD include extracellular amyloid plaques consisting of fibrillated Aβ and intraneuronal neurofibrillary tangles consisting of tau, and clinical manifestations consist of changes in personality and memory decline.[4] The amyloid cascade hypothesis states that excessive accumulation of Aβ initiates and drives the pathological changes in AD.[5] Specifically, amyloidogenic processing of APP and abnormal Aβ metabolism have been proposed to be two of the main causes of AD, supported by the mutations that cause increased Aβ production, in particular of the aggregation-prone Aβ42, leading to early-onset AD,[6] whereas a mutation that lowers Aβ production has a preventive effect.[7] A recent genetic meta-analysis implicated Aβ as a major risk factor associated with sporadic AD.[8] More than 130 agents are in clinical trials for treatment of AD, and soluble and/or aggregated Aβ is the most common target in disease modification trials.[9] There is a high rate of failure of compounds developed for AD, and many tested compounds, in particular antibodies, have poor permeability over the blood-brain barrier (BBB),[10] but recently a monoclonal antibody was approved by the US Food and Drug Administration for AD treatment.[11]

Molecular chaperones play a critical role in maintaining cellular protein homeostasis[12] and are attractive to harness for prevention or resolution of protein aggregation linked to neurodegenerative conditions.[13] BRICHOS is an about 100-residue domain found in 10 human protein families, and the name is derived from three of these, Bri2 (associated with dementia and brain amyloid), chondromodulin (chondrosarcoma), and prosurfactant protein C (proSP-C; interstitial lung disease and amyloid).[14] All BRICHOS-containing proproteins have well-conserved regions that are prone to form β strands.[15] The ability of BRICHOS to inhibit amyloid formation and toxicity was unraveled by the observation that mutations in the BRICHOS domain of proSP-C give rise to interstitial lung disease with amyloid deposits.[16,17] Recently it has been shown that this ability can be extended to amyloidogenic proteins and peptides that are not physiological clients.[18] Recombinant human (rh) BRICHOS can inhibit Aβ40 and Aβ42 fibril formation, and it also prevents neurotoxicity of Aβ42 in hippocampal slice preparations and in a *Drosophila melanogaster* fly model.[19–22] The neurotoxicity of Aβ42 is prevented by a unique mechanism: rh BRICHOS efficiently blocks monomer-dependent secondary nucleation on the Aβ42 fibril surface, the kinetic step

Received 3 February 2022; accepted 12 August 2022; https://doi.org/10.1016/j.ymthe.2022.08.010.

Correspondence: Jan Johansson, Department of Biosciences and Nutrition, Karolinska Institutet, Neo, 141 83 Huddinge, Sweden.
E-mail: janne.johansson@ki.se

This is an open access article under the CC BY license (http://creativecommons.org/licenses/by/4.0/).

that generates a major part of new nucleation units that can convert to neurotoxic Aβ42 oligomers.[23–25] BRICHOS can also rescue already established Aβ42-induced deterioration of hippocampal neural network activity *in vitro*.[26] Although not expected considering the size and polarity of the BRICHOS domain, intravenously injected rh Bri2 BRICHOS without addition of any targeting tag passes the BBB in wild-type mice.[27,28]

The ability of rh Bri2 BRICHOS to prevent Aβ42 oligomer mediated neurotoxicity *in vitro* and to pass the BBB warrant evaluation of its potential ability to improve AD-like features in animal models, which is the topic of the current study. Previous studies have demonstrated that rh Bri2 BRICHOS is distributed between different quaternary structures, where monomers are most potent in preventing Aβ42 oligomer generation and neurotoxicity, and they also pass the BBB more efficiently than larger oligomers.[28,29] Therefore, we generated a single point mutant (R221E) of rh Bri2 BRICHOS, which was designed to interfere with intersubunit contacts and, thus, stabilize the monomer. Rh Bri2 BRICHOS R221E was found to be overall more efficient than wild-type (WT) rh Bri2 BRICHOS against Aβ42-mediated neurotoxicity in mouse hippocampal slices *ex vivo*.[30] The corresponding mutation in proSP-C BRICHOS resulted in stable monomers that bind to small, secondary nucleation-competent Aβ42 oligomers.[25] We used two different *App* knockin AD mouse models that have different genetic designs and pathology profiles[31] in the current study. One model ($App^{NL\text{-}F}$ mice) harbors the Swedish (KM670/671NL) and Beyreuther/Iberian (I716F) mutations and develops Aβ plaque pathology, astrogliosis, and microgliosis starting from an age of about 9–12 months. The other model ($App^{NL\text{-}G\text{-}F}$ mice) in addition carries the Arctic mutation (E693G) and develops AD-like pathology features from an age of about 2–4 months because of the more aggressive fibril formation of Arctic Aβ. Both mouse models thus feature robust Aβ pathology, neuroinflammation, and synaptic alterations, whereas APP levels remain physiological, but $App^{NL\text{-}G\text{-}F}$ mice show more pronounced behavioral impairments.[31–33]

We treated $App^{NL\text{-}G\text{-}F}$ mice from the age of 3 months and $App^{NL\text{-}F}$ mice from the age of 19 months with intravenous injections of rh Bri2 BRICHOS R221E monomer for 10–12 weeks. Effects on all analyzed features, including improvements in cognitive functions, were found in $App^{NL\text{-}G\text{-}F}$ mice, whereas we found overall smaller but significant effects on plaque burden and astrocyte and microglia responses in $App^{NL\text{-}F}$ mice. We also found that *in vivo* effects of BRICHOS treatment correlate with amounts of Bri2 BRICHOS detected in brain sections after the end of treatment.

RESULTS

Rh Bri2 BRICHOS R221E effects on Arctic Aβ42 fibril formation *in vitro*, serum half-life, and BBB passage in mice

Rh Bri2 BRICHOS R221E monomers were produced and purified as described previously.[30] SDS-PAGE under reducing and non-reducing conditions showed that the preparations were pure, and the somewhat faster migration under non-reducing conditions (Figure 1A) confirmed the presence of the conserved disulfide bridge. Effects of rh Bri2 BRICHOS R221E on WT Aβ42 fibril formation and the mechanism involved have been reported.[30] Treatment attempts in $App^{NL\text{-}G\text{-}F}$ mice require that rh Bri2 BRICHOS R221E also affects Arctic Aβ42 ($Aβ42^{Arc}$) fibril formation. $Aβ42^{Arc}$ monomers at a concentration of 4.5 μM aggregated into amyloid fibrils with typical sigmoidal behavior and a half-time of 0.3 ± 0.04 h (Figure 1B), which is much faster than WT Aβ42.[34] The rh Bri2 BRICHOS R221E monomers showed a dose-dependent progressive reduction of $Aβ42^{Arc}$ fibril formation rate already at substoichiometric concentrations (Figure 1B). To determine rh Bri2 BRICHOS R221E passage over the BBB and its serum half-life, rh Bri2 BRICHOS R221E monomers at a dose of 10 or 20 mg/kg were injected intravenously into C57BL/6NTac mice, whereas control mice received PBS, and half-life in serum and BBB permeability were evaluated from western blots of serum and brain homogenates, respectively. *In vivo*, the serum half-life of rh Bri2 BRICHOS R221E monomers is about 55 ± 15 min (Figure 1C), which is somewhat longer than the half-life of WT rh Bri2 BRICHOS monomers, dimers, or oligomers (around 30–40 min).[28] Rh Bri2 BRICHOS R221E could be detected in brain homogenates by western blotting 2 h after injection (Figure 1D). Comparison of amounts detected in brain homogenates by western blot indicates that rh Bri2 BRICHOS R221E permeates the BBB to a similar extent as rh WT Bri2 BRICHOS linked to an AU1 tag for detection (Figure 1E).[28] As observed previously for WT rh Bri2 BRICHOS,[28] there was some variability in the amounts of rh Bri2 BRICHOS R221E detected by western blot of brain homogenates (Figures 1D and 1E). Therefore, we analyzed BBB passage of rh Bri2 BRICHOS R221E by staining brain sections from treated $App^{NL\text{-}G\text{-}F}$ and $App^{NL\text{-}F}$ mice for Bri2 BRICHOS and Aβ (Figure 2). Interpretation of this experiment is complicated by the fact that endogenous mouse Bri2 BRICHOS is also stained.[28] Rh Bri2 BRICHOS R221E-treated mice showed more abundant overall Bri2 BRICHOS staining in the brain tissue, including neurons (Figures 2A and 2D, red/pink) and around Aβ plaques (Figures 2A and 2D, blue/green) compared with PBS-treated mice. There is a significant increase in overall Bri2 BRICHOS staining, analyzed by mean intensity of Bri2 BRICHOS (red/pink) between PBS- and rh Bri2 BRICHOS R221E-treated samples in $App^{NL\text{-}G\text{-}F}$ and $App^{NL\text{-}F}$ mice (Figures 2B, 2C, 2E, and 2F). These results strongly support the hypothesis that intravenously injected rh Bri2 BRICHOS R221E crosses the BBB in mice. Further studies are warranted to explore whether upregulation of endogenous Bri2 expression can contribute to the increased Bri2 BRICHOS staining after rh Bri2 BRICHOS R221E treatment.

Treatment of $App^{NL\text{-}G\text{-}F}$ and $App^{NL\text{-}F}$ mice with intravenous rh Bri2 BRICHOS is well tolerated

We administered rh Bri2 BRICHOS R221E at a dose of 10 mg/kg to $App^{NL\text{-}G\text{-}F}$ mice from 3 months of age every fifth day for 12 weeks, totaling 17 injections (Figure 3A), and $APP^{NL\text{-}F}$ mice were given a total of 20 injections of rh Bri2 BRICHOS R221E monomer at a dose of 20 mg/kg during a 10-week period from 19 months of age (Figure 3B). For $App^{NL\text{-}G\text{-}F}$ mice, the treatment starts at about the age when AD-like pathology is first observed.[31] In contrast, treatment of $App^{NL\text{-}F}$ mice was started at least 4 months after AD-like pathology is well established.[31] For both mouse models, the treatment period

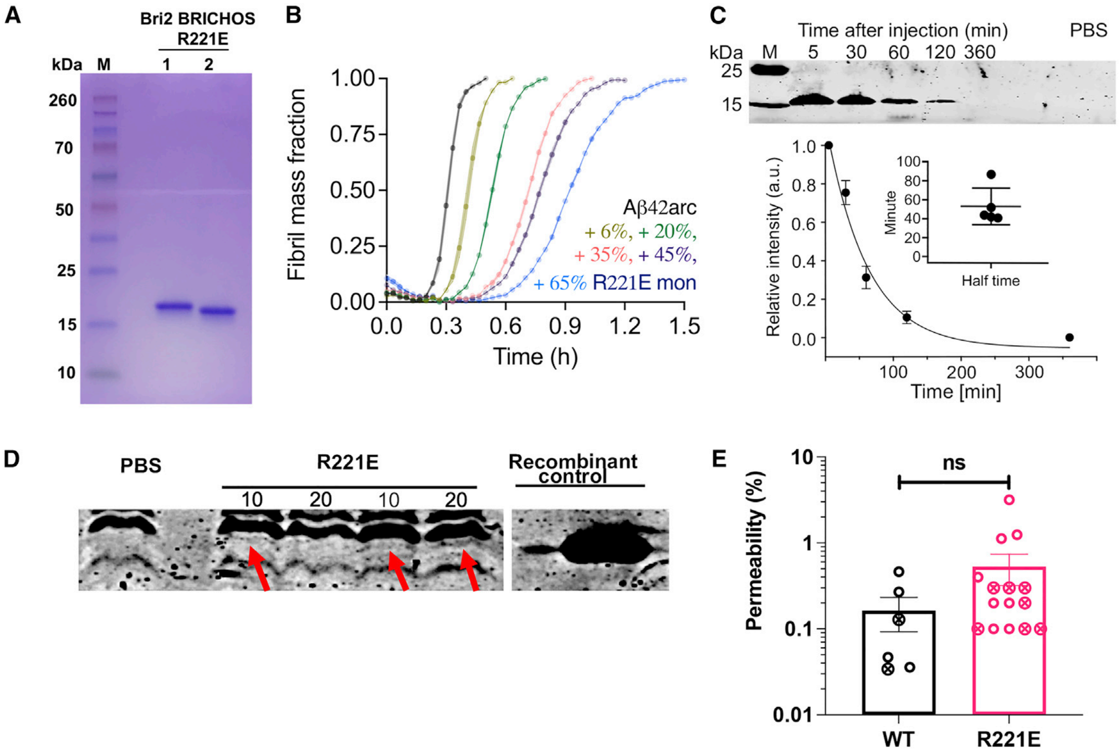

Figure 1. Recombinant human Bri2 BRICHOS R221E monomer purity, effects on Aβ42^{Arc} *in vitro,* serum half-life, and BBB passage

(A) rh Bri2 BRICHOS R221E monomer analyzed by reducing (lane 1) and non-reducing (lane 2) SDS-PAGE.

(B) Aggregation kinetics of Aβ42^{Arc} alone and in the presence of different molar ratios of rh Bri2 BRICHOS R221E monomers.

(C) rh Bri2 BRICHOS R221E levels in serum up to 6 h after injection and half-life in serum derived from analysis of intensities in western blots. Data are shown as mean ± SEM (n = 5).

(D) Representative western blot for rh Bri2 BRICHOS R221E in brain homogenates 2 h after i.v. injection of 10 or 20 mg/kg of body weight of rh Bri2 BRICHOS R221E (as indicated), PBS, or pure recombinant protein. The gap between the rightmost R221E lane and the recombinant control lane denotes that they are not adjacent, but the samples were run on the same gel. Red arrows indicate Bri2 BRICHOS R221E bands.

(E) Histogram showing permeability over the BBB, expressed as percent protein detected by western blots of brain homogenates, of the total amount of protein injected i.v., after injection of 10 (open circles) or 20 (crossed circles) mg/kg of rh WT Bri2 BRICHOS-AU1 (detected using an anti-AU1 antibody) or rh Bri2 BRICHOS R221E (detected using an anti-Bri2 BRICHOS antibody). Quantification was made from band intensities compared with intensities of known amounts of recombinant protein controls. The three highest data points for rh Bri2 BRICHOS R221E are replicates from the same mouse, and all other data points are from different mice. Ns, not significant. Data are shown as mean ± SEM.

was followed by behavioral testing for 2–4 weeks; thereafter the mice were sacrificed and the brains analyzed for Aβ plaque burden, astrogliosis, and microgliosis (Figures 3A and 3B).

Treatment of *App*$^{NL-G-F}$ or *App*$^{NL-F}$ mice with intravenous rh Bri2 BRICHOS R221E resulted in no macroscopic signs of unwanted side effects or weight loss compared with PBS-injected controls (Figures 3C and 3D). For *App*$^{NL-G-F}$ mice, no differences in mean activity or velocity were seen between rh Bri2 BRICHOS R221E-treated mice and PBS-treated controls, and treatment of *App*$^{NL-F}$ mice with rh Bri2 BRICHOS R221E somewhat improved physical activity compared with PBS-injected *App*$^{NL-F}$ mice (data not shown). These findings further support the hypothesis that treatment with rh Bri2 BRICHOS R221E is without negative side effects. Rh Bri2 BRICHOS R221E is 97% identical to the mouse counterpart, making it possible that no immunological response was evoked, which is in line with the absence of detectable negative reactions.

Improved recognition and working memory in rh Bri2 BRICHOS R221E-treated App^{NL-G-F} mice

App^{NL-G-F} knockin mice, which carry the Arctic mutation in addition to the Swedish and Iberian mutations, show a stronger and earlier AD-like pathology, including behavior deficits, compared with *App*$^{NL-F}$ knockin mice[31,32] (Figures 3A and 3B). Recognition memory was evaluated by novel object recognition (NOR) in rh Bri2 BRICHOS R221E- and PBS-treated *App*$^{NL-G-F}$ mice. Significant improvement in discrimination ability was seen upon rh Bri2 BRICHOS R221E treatment compared with PBS controls (Figure 4A). This indicates that rh Bri2 BRICHOS R221E-treated *App*$^{NL-G-F}$ mice exhibited improved recognition memory compared with their PBS counterparts. A Y maze test was used to evaluate short-term working memory in PBS- and rh Bri2 BRICHOS R221E-treated *App*$^{NL-G-F}$ mice, and the results showed improvements in the number of spontaneous alternations in rh Bri2 BRICHOS R221E-administered mice

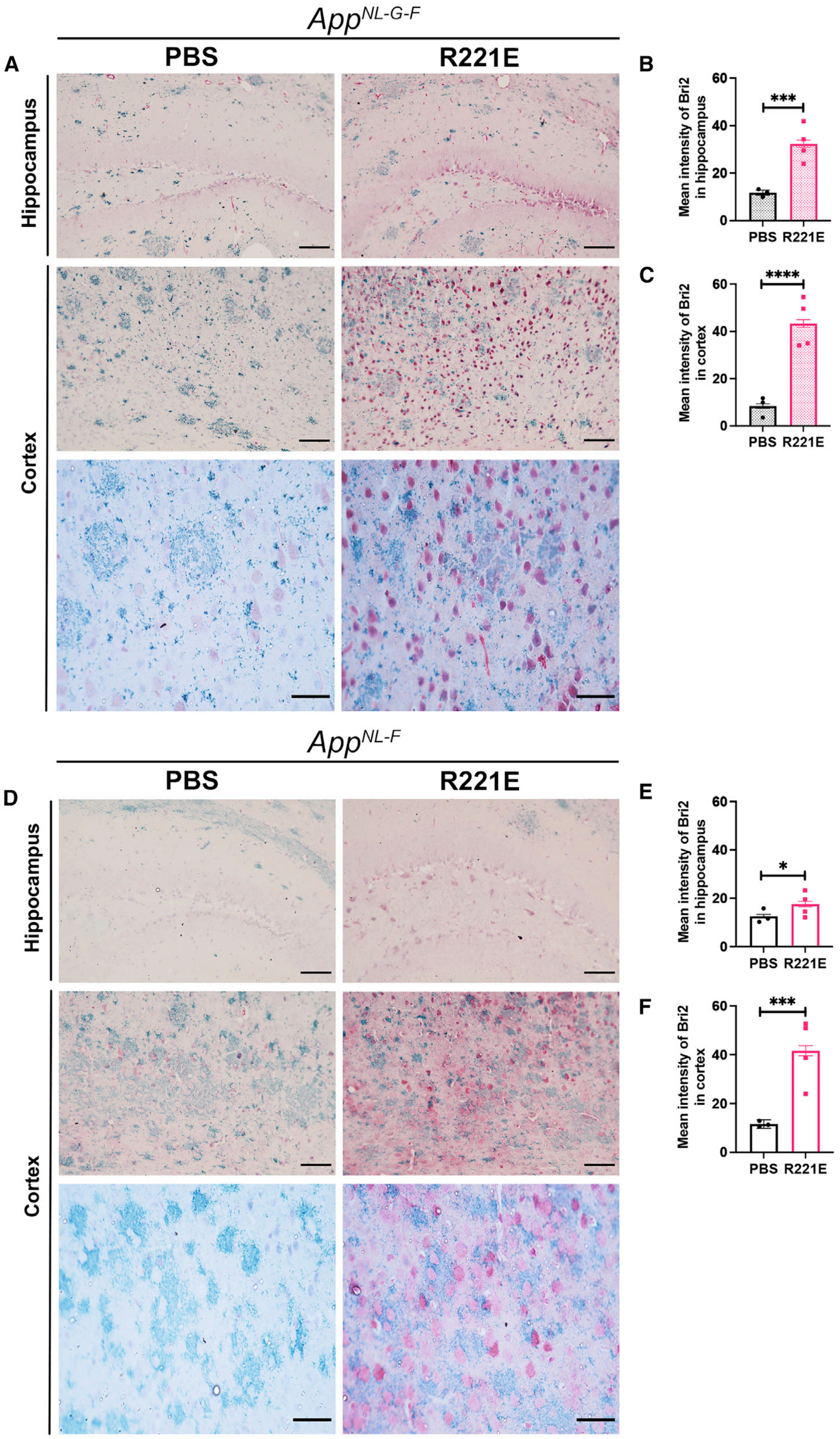

(legend on next page)

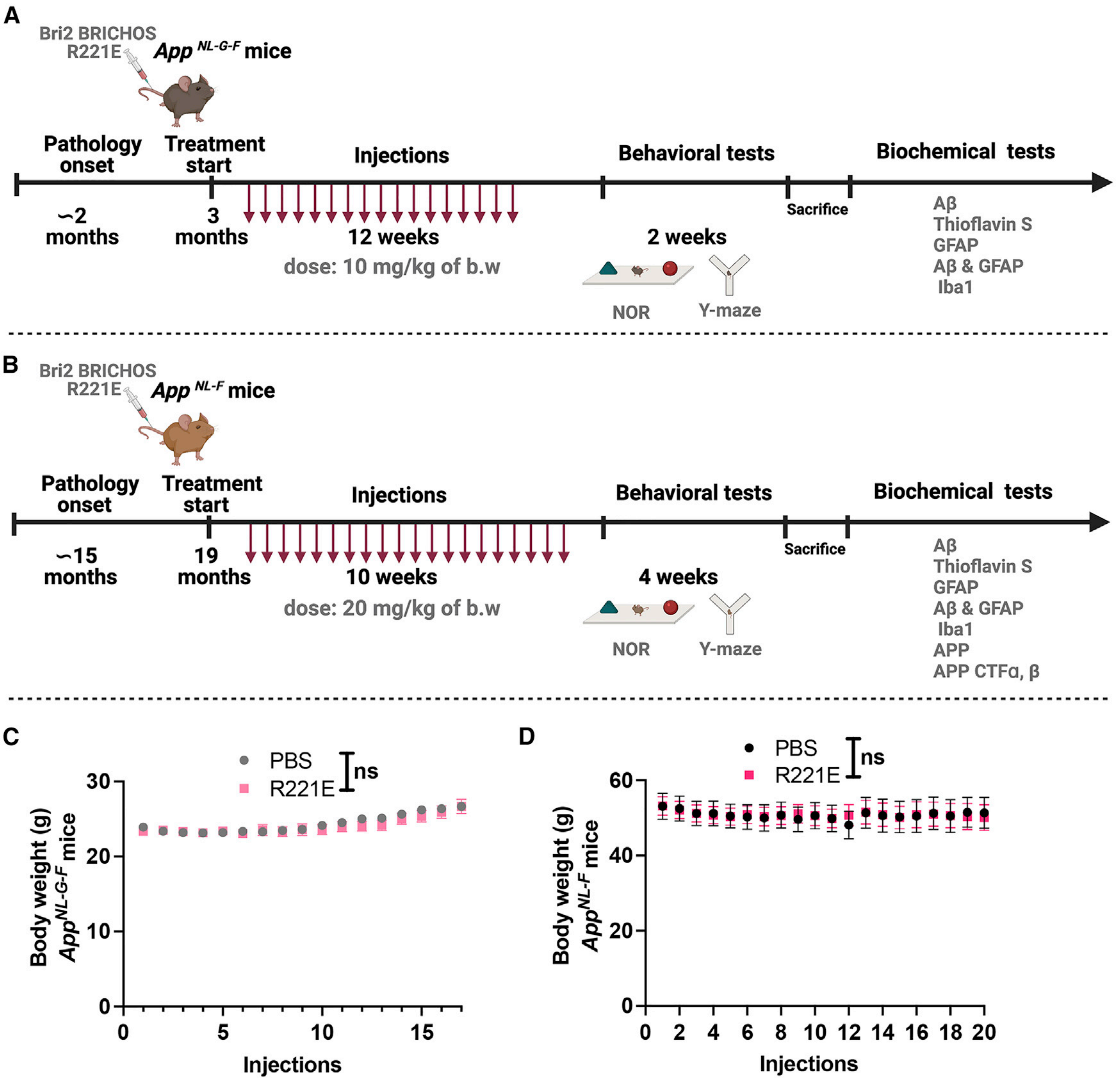

Figure 3. Study design and body weight of App^{NL-G-F} and App^{NL-F} mice

(A and B) Schematic of the overall design of the study, with time points for treatments and analyses indicated. b.w., body weight; NOR, novel object recognition. The lengths of the time lines are not linear to the durations of intervals.

(C and D) B.w. of PBS or rh Bri2 BRICHOS R221E-treated App^{NL-G-F} and App^{NL-F} mice before each injection during the entire treatment periods. Data are represented as mean ± SEM. Multiple unpaired parametric t test was used to calculate p values for b.w. analysis. (A) and (B) were created using BioRender.

compared with PBS control mice (Figure 4B). Although the improvement was modest, the results support an improved working memory response upon rh Bri2 BRICHOS R221E treatment.

Attenuation of Aβ plaque burden in App^{NL-G-F} mice with rh Bri2 BRICHOS R221E treatment

We stained brain slices with thioflavin S dye or Aβ antibody to analyze plaque burden in rh Bri2 BRICHOS R221E- and PBS-treated

Figure 2. Bri2 BRICHOS in App^{NL-G-F} and App^{NL-F} mouse brains after repeated injections of rh Bri2 BRICHOS R221E

(A–F) Representative sections (A and D) double stained with anti-Bri2 BRICHOS antibody (red/pink) and 82E1 anti-Aβ antibody (green) counterstained with hematoxylin Mayer (blue) in rh Bri2 BRICHOS R221E- or PBS-treated App^{NL-G-F} and App^{NL-F} mice. Histograms represent mean intensity of Bri2 BRICHOS in the hippocampus (B and E) and cortex (C and F) of App^{NL-G-F} (B and C) and App^{NL-F} (E and F) mice. Data are shown as mean ± SEM (n = 3–4 mice/group, 4 histological sections from each mouse analyzed). Unpaired parametric two-tailed t test was used to calculate p values. Scale bars represent 400 μm (top four panels in A and D) and 100 μm (bottom two panels in A and D). *p < 0.05, **p < 0.01, ***p < 0.001, ****p < 0.0001.

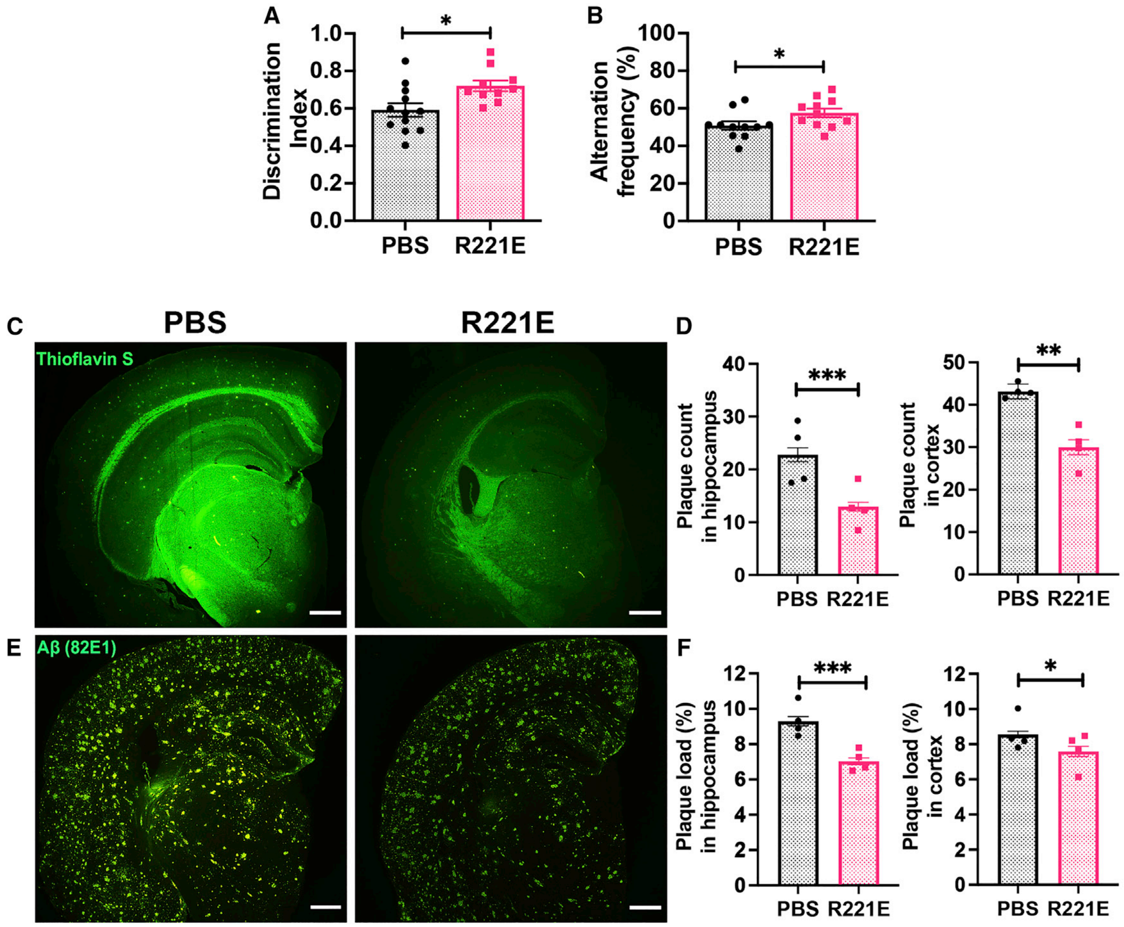

Figure 4. Improvements in recognition and working memory and reduced plaque count and plaque load with rh Bri2 BRICHOS R221E treatment in *App*$^{NL\text{-}G\text{-}F}$ mice

(A–F) Discrimination index (A) assessed during the NOR test to evaluate the recognition memory response in PBS- and rh Bri2 BRICHOS R221E-treated *App*$^{NL\text{-}G\text{-}F}$ mice. The alternation frequency (B) during the Y maze test was assessed for working memory response in PBS- and rh Bri2 BRICHOS R221E-treated *App*$^{NL\text{-}G\text{-}F}$ mice (n = 11–12 mice/group). Shown are representative images of PBS and rh Bri2 BRICHOS R221E-treated mice, stained with thioflavin S (C) or immunostained with 82E1 Aβ antibody (E) for amyloid plaques and histograms representing plaque count (D) analyzed from thioflavin S-stained brain slices and plaque load (F) analyzed from 82E1 anti-Aβ antibody-stained brain slices in the hippocampus and cortex regions. Data are represented as mean ± SEM (n = 4 mice/group, 4 histological sections per mouse for immunostaining). Unpaired parametric two-tailed t test was used to calculate the p values. The scale bar represents 500 μm. A 0.40 value in the rh Bri2 BRICHOS R221E group was removed as an outlier for (A). *p < 0.05, **p < 0.01, ***p < 0.001.

mice. Rh Bri2 BRICHOS R221E-treated *App*$^{NL\text{-}G\text{-}F}$ mice showed a distinct reduction in Aβ plaque count, as analyzed by thioflavin S staining (Figures 4C and 4D), in the hippocampus and cortex. Aβ plaque load, analyzed using Aβ antibody immunostaining, was reduced in rh Bri2 BRICHOS R221E-treated mice in hippocampus and cortex regions (Figures 4E and 4F).

Reduced astrogliosis and microgliosis in App$^{NL\text{-}G\text{-}F}$ mice after rh Bri2 BRICHOS R221E treatment

Astrogliosis was evaluated by analyzing the expression of an astrocyte marker, glial fibrillary acidic protein (GFAP), by immunostaining of brain slices. In *App*$^{NL\text{-}G\text{-}F}$ mice treated with rh Bri2 BRICHOS R221E, we observed a reduction in GFAP-positive astrocytes in hippocampus and cortex regions compared with PBS controls (Figures 5A–5C).

Areas with GFAP-positive astrocytes often appeared as circular clusters, indicating that they may surround the plaques. To address this, we double-stained brain slices for GFAP and Aβ, and the results show abundant localization of GFAP-positive cells around Aβ plaques. The extent of colocalization was reduced in rh Bri2 BRICHOS R221E-treated *App*$^{NL\text{-}G\text{-}F}$ mice compared with PBS controls in hippocampus and cortex regions (Figures 5D–5F).

Evidence from animal and human studies suggests involvement of microglia in the pathological cascade of AD, and we used the

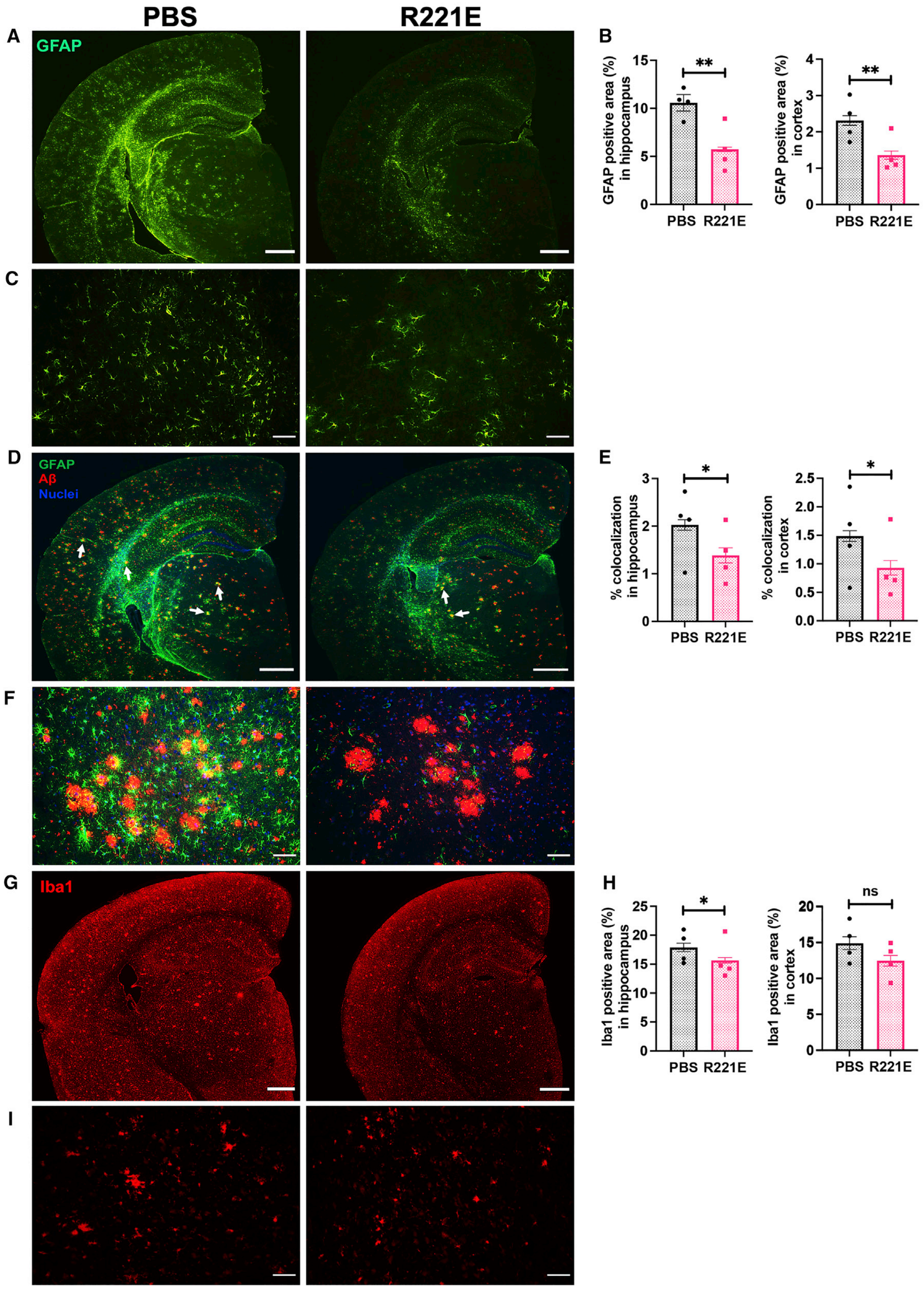

(legend on next page)

microglial activation marker ionized calcium-binding adapter molecule 1 (Iba1)[35,36] to analyze rh Bri2 BRICHOS R221E- and PBS-treated App^{NL-G-F} mice. A significant reduction in Iba1-positive microglia upon rh Bri2 BRICHOS R221E treatment compared with PBS controls was seen in the hippocampus, whereas there was a trend toward a reduction in the cortex (Figures 5G–5I).

Effects of rh Bri2 BRICHOS R221E treatment on aged App^{NL-F} mice

Our results show that object recognition memory and short-term working memory are improved in App^{NL-G-F} mice treated with rh Bri2 BRICHOS R221E, paralleled by a robust reduction in Aβ plaque burden, astrocyte activation, and colocalization of GFAP and Aβ, whereas reduced microglia activation was observed in the hippocampus but not in the cortex. Treatment of App^{NL-G-F} mice commenced at about the same time as AD-relevant pathology started to develop, making the approach difficult to translate to the goal of treating individuals with AD with already established pathology. We therefore repeated that treatment schedule in App^{NL-F} mice but started the treatment at an age when AD pathology is already well established (Figure 3B). We also used a higher dose, 20 mg/kg intravenous rh Bri2 BRICHOS R221E instead of 10 mg/kg used in the App^{NL-G-F} mice, and the drug holiday period after the last dose until sacrifice was 4 weeks instead of 2 weeks (Figures 3A and 3B).

In App^{NL-F} mice, NOR memory and Y maze tests showed no detectable difference between the two treatment groups (Figures 6A and 6B). Literature data on App^{NL-F} knockin mice show comparatively modest behavioral and cognitive deficits compared with WT mice, also at old age, which could make detection of behavioral improvements difficult in this mouse model.[31,32] However, we observed a lower discrimination index in the NOR test in 22-month-old App^{NL-F} mice than in 6-month-old App^{NL-G-F} mice (Figures 4A and 6A). This difference could be explained by the difference in age between the mouse models at the time of analyses, but it also suggests that the lack of effects on NOR memory after rh Bri2 BRICHOS R221E treatment in App^{NL-F} mice could be due to factors other than mild behavior and cognitive pathology in this mouse model.

Regarding plaque burden, however, rh Bri2 BRICHOS R221E-treated App^{NL-F} mice have lower thioflavin S-positive plaque counts (Figures 6C and 6D) and a somewhat lower Aβ-positive plaque load (Figures 6E and 6F) in the cortex compared with PBS controls, but in the hippocampus, no significant differences between the groups were found (Figures 6C–6F). To evaluate the possibility that rh Bri2 BRICHOS R221E treatment affects APP levels or processing, brain cortex homogenates were analyzed for the amounts of full-length APP and the ratio of APP derived C-terminal fragment α (CTFα) and CTFβ in rh Bri2 BRICHOS R221E-treated and PBS-treated control mice (Figure S1). No differences were seen in APP levels or ratio of APP processing products between rh Bri2 BRICHOS R221E- and PBS-treated mice, which supports the hypothesis that the reduced Aβ plaque burden (Figures 4 and 6) is the result of rh Bri2 BRICHOS R221E effects on Aβ fibril formation.

Regarding effects on gliosis in App^{NL-F} mice, GFAP immunostaining of brain slices showed that activated astrocytes were reduced in rh Bri2 BRICHOS R221E-treated mice compared with PBS controls in the cortex but not in the hippocampus (Figures 7A–7C). The extent of GFAP and Aβ colocalization was, however, reduced in rh Bri2 BRICHOS R221E-treated mice in cortex and hippocampus regions compared with PBS controls (Figures 7D–7F). Microgliosis in App^{NL-F} mice, as measured by Iba1 staining, was significantly reduced in the cortex, and a trend toward reduction was seen in the hippocampus after rh Bri2 BRICHOS R221E treatment compared with PBS controls (Figures 7G–7I).

DISCUSSION

Intravenous administration of rh Bri2 BRICHOS R221E for 10–12 weeks in *App* knockin mouse models results in apparent accumulation of the protein in the brain, including in neurons (Figure 2), and the treatment regimen is well tolerated (Figure 3) and improves multiple AD features in the mice (Figure 8). Treatment of App^{NL-G-F} mice with rh Bri2 BRICHOS R221E resulted in significant improvements in all AD-relevant features measured, whereas for App^{NL-F} mice no effects were seen on learning and memory, and reduced plaque burden and gliosis were only seen in the cortex (Figure 8). These differences can have several explanations, such as more aggressive development of AD pathology in App^{NL-G-F} mice, different doses of rh Bri2 BRICHOS R221E given, different durations between the last dose and biochemical analyses, as well as more time elapsed after AD-like pathology was established in App^{NL-F} mice until treatment was started compared with App^{NL-G-F} mice. The Aβ42 levels increase almost two orders of magnitude from 9 to 19 months of age in App^{NL-F} mice,[31] and it is likely that AD pathology features become treatment resistant with increasing age. The treatment efficacy differences between the two mouse models correlate well with the amount of Bri2 BRICHOS detected in brain tissue after the end of treatment; App^{NL-G-F} mice show more robust Bri2 BRICHOS staining than App^{NL-F} mice, in particular in the

Figure 5. Rh Bri2 BRICHOS R221E mitigates astrogliosis and microgliosis and reduces colocalization of Aβ and GFAP in App^{NL-G-F} mice

(A–I) Representative images of brain sections stained with anti-GFAP antibody (A and C) and histograms showing a GFAP-positive area in the hippocampus and cortex (B). Shown are representative merged images of brain sections double stained using anti-Aβ and anti-GFAP antibodies and counterstained with Hoechst nuclear stain (D and F) and histograms representing percent colocalization of Aβ and GFAP in the hippocampus and cortex regions (E). Also shown are representative images of brain sections stained with anti-Iba1 antibody (G and I) and histograms showing Iba1-positive areas in the hippocampus and cortex (H). Data are shown as mean ± SEM for PBS- and rh Bri2 BRICHOS R221E-treated App^{NL-G-F} mice (n = 4 mice/group, 4 histological sections per mouse). Unpaired parametric two-tailed t test was used to calculate the p values. Scale bars represent 500 μm (A, D, and G) and 20 μm (C, F, and I). White arrows in (D) identify examples of Aβ and GFAP colocalized spots visible in yellow. $^{*}p < 0.05$, $^{**}p < 0.01$.

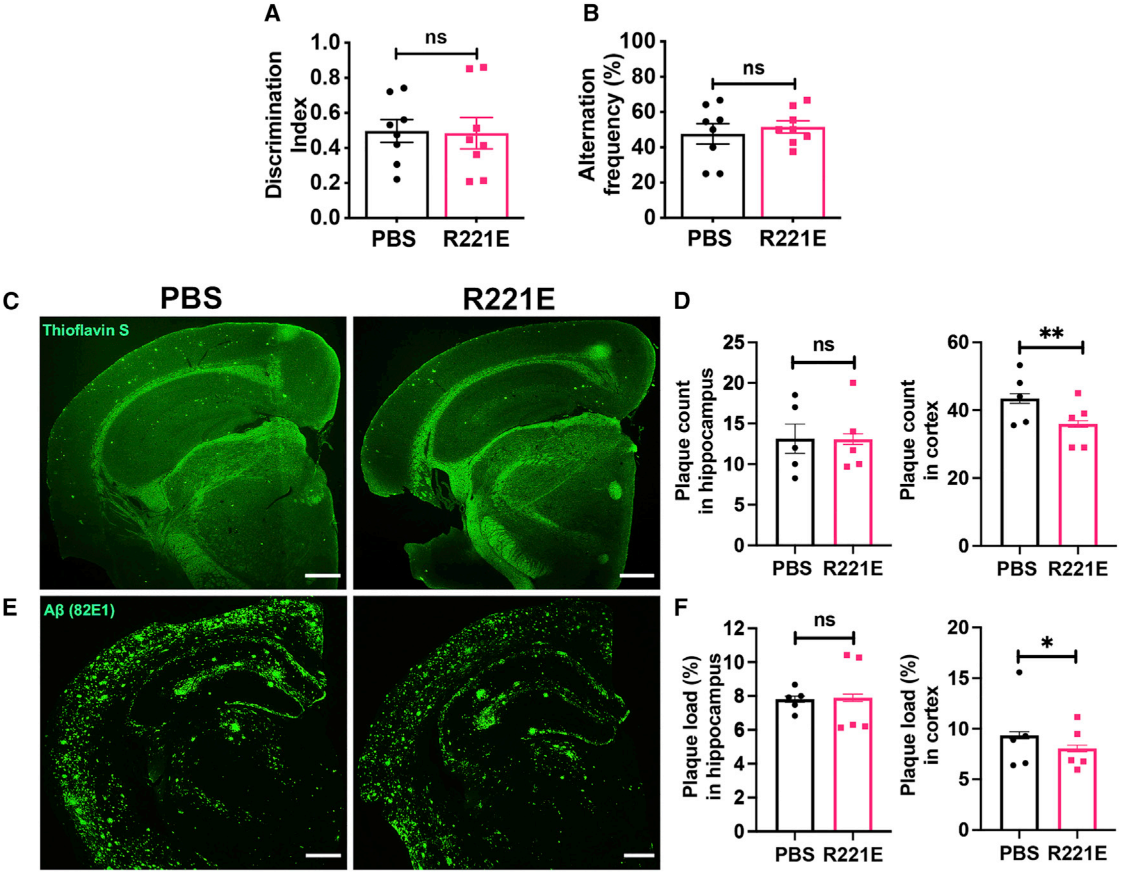

Figure 6. Reduced plaque counts and plaque load in the cortex region of the brain with no change in memory functions with rh Bri2 BRICHOS R221E treatment in *App^NL-F* mice

(A–F) Discrimination index (A) assessed during the NOR test to evaluate the recognition memory response in PBS- and rh Bri2 BRICHOS R221E-treated *App^NL-F* mice. The alternation frequency (B) during the Y maze test was assessed for working memory response in PBS- and rh Bri2 BRICHOS R221E-treated *App^NL-F* mice (n = 8 mice/group). Shown are representative images of PBS- and rh Bri2 BRICHOS R221E-treated mice, stained with thioflavin S (C) or immunostained with 82E1 anti-Aβ antibody (E) for amyloid plaques. Histograms represent plaque count analyzed from thioflavin S-stained brain slices (D) and plaque load analyzed from 82E1 anti-Aβ antibody-stained brain slices (F) in hippocampus and cortex regions (n = 5 mice/group, 4 histological sections per mouse). Data are represented as mean ± SEM. Unpaired parametric two-tailed t test was used to calculate the p values. Scale bars represent 500 μm. $^{*}p < 0.05$, $^{**}p < 0.01$.

hippocampus (Figure 2). *App^NL-F* mice received higher doses of rh Bri2 BRICHOS R221E, but, on the other hand, the time period between the last dose and biochemical analyses was longer than in *App^NL-G-F* mice (Figure 3). In *App^NL-F* mice, Bri2 BRICHOS staining is more pronounced in the cortex than in the hippocampus (Figure 2), which correlates with the biochemical effects seen in the cortex region in this mouse model (Figure 8).

A growing body of evidence indicates that inflammatory reactions have a crucial role in the pathogenesis of AD, and reactive astrocytes and microglia are commonly found in proximity of Aβ plaques in AD.[37–40] A recent study in *App^NL-G-F* mice and humans using spatial transcriptomics and *in situ* sequencing showed that specific genes are induced in the tissue surrounding plaques.[41] With this in mind, it is interesting that one feature that was robustly affected in *App^NL-G-F* and *App^NL-F* mice in our study was astrocyte activation in the close vicinity of Aβ plaques (Figures 5D–5F, 7D–7F, and 8). This raises the possibility that plaques provide surfaces for generation of soluble toxic Aβ species and that rh Bri2 BRICHOS R221E efficiently interferes with the generation of such species.

Adenovirus-mediated transgenic overexpression of Bri2 BRICHOS in transgenic APP/presenilin 1 mice for 8 months had qualitatively similar effects on Aβ plaque load and astrogliosis[42] as the effects observed here after intravenous administration of rh Bri2 BRICHOS R221E to *App^NL-G-F* mice and *App^NL-F* mice. These observations support the hypothesis that the treatment effects seen here are mediated by the BRICHOS domain. For anti-Aβ antibodies used in clinical trials, reported effects in transgenic AD mouse models are limited to reduced plaque burden, but passive immunization against Aβ in general has shown improvement in behavior as well.[43,44] The results for rh Bri2 BRICHOS R221E and monoclonal antibodies have

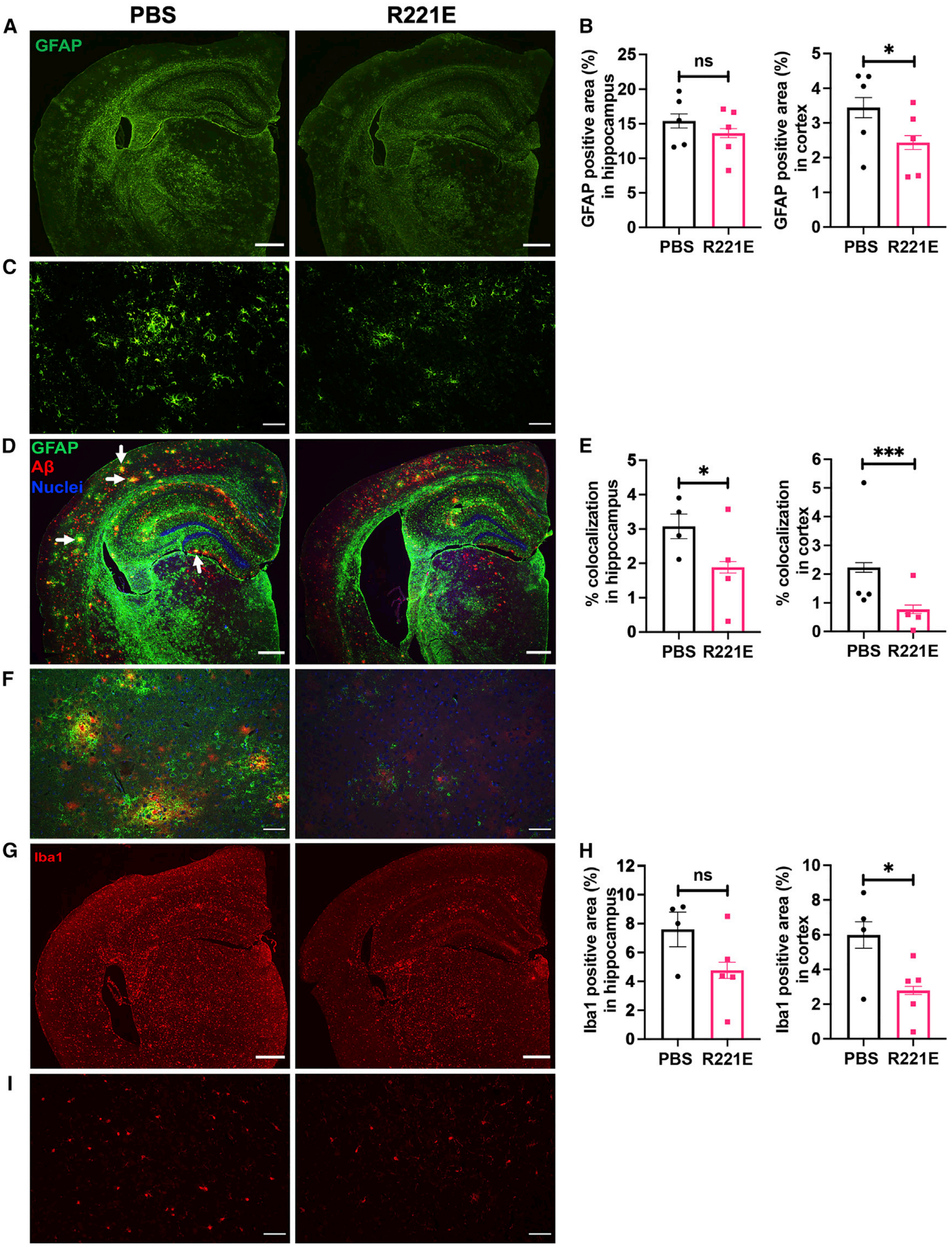

Figure 7. Rh Bri2 BRICHOS R221E mitigates astrogliosis and microgliosis and reduces colocalization of Aβ and GFAP in *App*$^{NL-F}$ mice

(A–I) Representative images of brain sections stained with anti-GFAP antibody (A and C) and histograms showing GFAP-positive areas in the hippocampus and cortex with n = 5 mice/group, 4 histological sections per mouse (B). Shown are representative merged images of brain sections double stained using anti-Aβ and anti-GFAP antibodies and counterstained with Hoechst nuclear stain (D and F) and histograms representing percent colocalization of Aβ and GFAP in hippocampus and cortex regions with n = 4

(legend continued on next page)

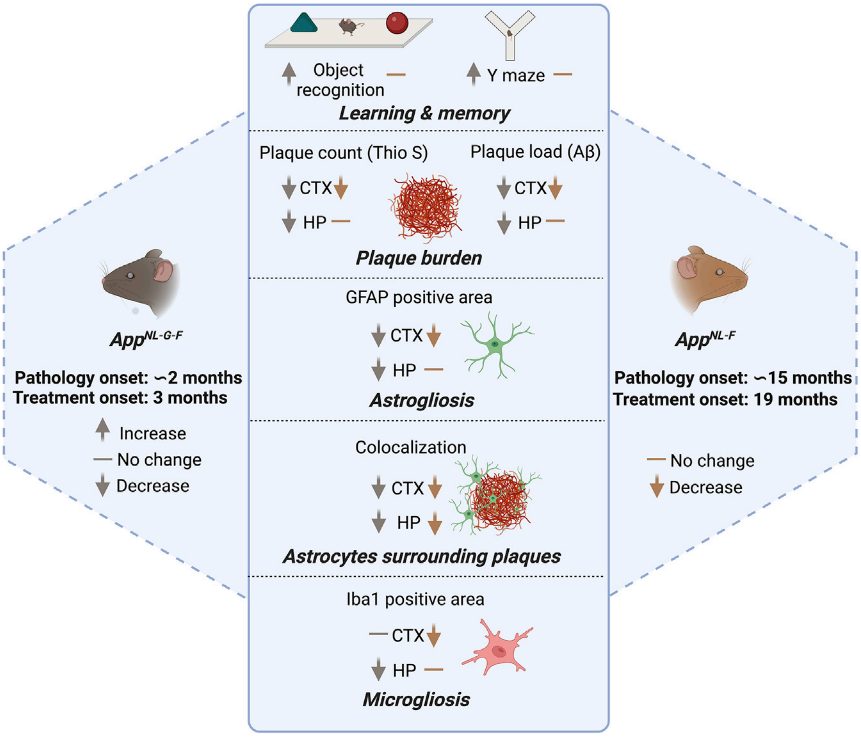

Figure 8. Overview of results of rh Bri2 BRICHOS R221E treatment of two *App* knockin AD mouse models

Shown is a schematic of rh Bri2 BRICHOS R221E treatment and effects obtained after treatment in *App*$^{NL\text{-}G\text{-}F}$ and *App*$^{NL\text{-}F}$ mice. CTX, cortex; HP, hippocampus. Arrows and signs indicate an increase, decrease, or no change in the features measured after rh Bri2 BRICHOS R221E treatment compared with PBS-treated controls. The figure was created using BioRender.

been obtained using different treatment schemes and different AD mouse models, and they can therefore not be directly compared. The recently reported higher efficiency of BRICHOS in reducing generation of Aβ42 oligomers *in vitro* compared with antibodies used in AD clinical trials [45] indicate that further comparative studies using *in vivo* models are warranted. Several studies have shown that the BRICHOS domain potently blocks Aβ42-mediated reduction of γ oscillations in mouse hippocampal slices *in vitro*.[19,21,24,29,30] γ Oscillations are reduced in individuals with AD and in *App*$^{NL\text{-}G\text{-}F}$ mice, and γ stimulation ameliorates AD-associated pathology and improves cognition in AD mouse models.[46–49]

This is the first study showing the effects of intravenous treatment with rh Bri2 BRICHOS R221E in AD mouse models, and several features were studied, including behavior, plaque burden, and gliosis, but a few parameters were included for each feature (Figure 8). This limitation can be addressed in future studies, in which additional mechanisms potentially mediated by the BRICHOS domain *in vivo* can be revealed. For example, single-cell transcriptome sequencing[50] of BRICHOS-treated mice compared with controls can give valuable information on gene products and pathways that are associated with the observed reduction in AD-like pathology.

MATERIALS AND METHODS

Study design

Female *App*$^{NL\text{-}G\text{-}F}$ mice were treated for 12 weeks with intravenous injections of PBS or rh Bri2 BRICHOS R221E (10 mg/kg, n = 12/group) every fifth day (total of 17 injections) starting from an age of 3 months. One mouse in the R221E group died before the last injection. Female *App*$^{NL\text{-}F}$ mice received PBS or rh Bri2 BRICHOS R221E (20 mg/kg of body weight, n = 10/group) injections intravenously twice every week for 10 weeks from the age of 19 months. Two mice from each group died after receiving the 14th–16th injections. All mice were caged in groups of 3–5 individuals, and the light-dark condition was 12:12 h (lights on at 08:00). Mice were randomly divided for PBS or rh Bri2 BRICHOS R221E administration. The mice were anesthetized using 2%–4% isoflurane and injected with PBS or rh Bri2 BRICHOS R221E with slow infusion. The study used a battery of behavior and biochemical assays to determine the treatment effects (Figures 2A and 2B). The experimenters assessing the behavior and biochemical assays were blinded to the intervention group. The behavior experiments were conducted during the light phase (10:00–18:00). The numbers for all biological repeats are given in the respective section. Outliers were detected using the Rout method (Q = 1%) or Grubbs method (alpha = 0.2) using Prism 8 (GraphPad, CA, USA) and removed as reported in the respective figure legends. All animal handling and experimental procedures were carried out in the animal facility, Huddinge Campus, Karolinska Institutet, according to local ethics guidelines and approved by Södra Stockholm's Djurförsöksetiska Nämnd (dnr S 6–15) and Linköping's animal ethical board (ID 855).

Recombinant protein expression and purification

A gene fragment encoding rh NT*-Bri2 BRICHOS R221E fusion protein, where NT* is a solubility tag[51] followed by human Bri2 residues 113–231, was cloned and expressed as described.[30] The protein was

mice/group, 4 histological sections per mouse (E). White arrows in (D) identify examples of yellow (i.e., Aβ and GFAP colocalized) spots. Also shown are representative images of brain sections stained with anti-Iba1 antibody (G and I) and histograms showing Iba1-positive areas in the hippocampus and cortex (n = 4–5 mice/group, 4 histological sections per mouse) (H). Data are shown as mean ± *SEM*. Unpaired parametric two-tailed t test was used to calculate the p value for all analyses. Scale bars represent 500 μm (A, D, and G) and 20 μm (C, F, and I). $^{*}p < 0.05$, $^{***}p < 0.001$.

expressed in Shuffle T7-competent *Escherichia coli* cells that were grown in lysogeny broth (LB) medium supplemented with 15 μg/mL kanamycin at 30°C. When optical density 600 (OD_{600}) reached ~0.9, the temperature was lowered to 20°C, and overnight protein expression was induced by addition of 0.5 mM isopropyl β-D-1-thiogalactopyranoside (IPTG). Cells were harvested by centrifugation (3,000 × *g*, 4°C), and cell pellets were re-suspended in 20 mM Tris-HCl (pH 8.0), followed by 5-min sonication (2 s on, 2 s off, 65% power, on ice). The lysate was centrifuged (24,000 × *g*, 4°C) for 30 min, and the supernatant containing the target protein was purified with an immobilized metal affinity chromatography (IMAC) column (Ni Sepharose 6 Fast Flow; GE Healthcare, UK) equilibrated with 20 mM Tris-HCl (pH 8.0). The fusion protein was eluted with 300 mM imidazole in 20 mM Tris-HCl (pH 8.0) and dialyzed (regenerated cellulose [RC], 6- 8-kDa membrane; Spectrum Lab) against 20 mM Tris-HCl (pH 8.0) overnight in a cold room. To remove the His_6-NT* part, the fusion protein was incubated with thrombin (1:600 enzyme-to-substrate weight ratio, Merck) for 72 h at 4°C, and then the proteins were re-applied onto a second IMAC column to remove the His_6-NT* tag. After concentration using a 10-kDa Vivaspin 20 column (GE Healthcare, UK) at 4,000 × *g* (4°C), the monomeric rh Bri2 BRICHOS R221E fractions were isolated by Superdex 75 PG column (GE Healthcare, UK) using an ÄKTA system (GE Healthcare, UK). The final monomeric proteins were dialyzed against filtered and autoclaved 1× PBS (pH 7.4), concentrated in a 5-kDa Vivaspin 20 column (GE Healthcare, UK) to the desired concentration, and filtered through a 0.2-μm Millex-GV filter (Merck Millipore, Ireland). The final purity of rh monomeric Bri2 BRICHOS R221E was evaluated by Coomassie staining after SDS-PAGE.

Aβ42^{Arc} monomer preparation and fibrillization

The recombinant Aβ(1–42) E22G mutant (Aβ42^{Arc}) was produced in BL21(DE3) *E. coli* cells and purified using the same protocol as for WT Aβ42.[34] Briefly, Aβ42^{Arc} was expressed fused to the solubility tag NT*. Immobilized-metal affinity chromatography-purified NT*-Aβ42^{Arc} was cleaved by tobacco etch virus (TEV) proteinase in a cold room overnight and lyophilized. The lyophilized powder was re-dissolved in 7 M Gdn-HCl, and Aβ42^{Arc} monomers were isolated by a Superdex 30 column 26/600 (GE Healthcare, UK) in 20 mM sodium phosphate (pH 8.0) with 0.2 mM EDTA. The concentration of monomeric Aβ42^{Arc} was calculated with an extinction coefficient of 1,424 M^{-1} cm^{-1} for ($A_{280}-A_{300}$).

For testing effects on Aβ42^{Arc} fibril formation, 20 μL solution containing 10 μM thioflavin T (ThT), 4.5 μM Aβ42^{Arc} monomer, and different concentrations of rh Bri2 R221E monomers at molar ratios of 0%, 6%, 20%, 35%, 45%, or 65% relative to the Aβ42^{Arc} monomer were added to each well of half-area 384-well microplates (Corning Glass 3766, USA) and incubated at 37°C under quiescent conditions. ThT fluorescence was recorded using a 440-nm excitation filter and 480-nm emission filter with a microplate reader (FLUOStar Galaxy; BMG Labtech, Offenberg, Germany). For all experiments, fibrillization traces were normalized and averaged using four replicates. A detailed analysis of the effects of rh Bri2 BRICHOS on Aβ42^{Arc} fibril formation was recently published.[52]

Analysis of serum half-life and BBB passage of rh Bri2 BRICHOS R221E

Three-month-old C57BL/6NTac (Taconic, Denmark) mice and 11-month-old C57BL/6J mice (Janvier Labs, France) were kept under controlled humidity and temperature on a 12-h light-dark cycle and group housed (seven per cage) with food and water available *ad libitum*. 3 mice received a single intravenous (i.v.) injection of rh Bri2 BRICHOS R221E monomers, 20 mg/kg, or an equal volume of PBS, into the lateral tail vein by using a 0.3-mL syringe with a 30G needle. Before the injections, the mice were placed in a single cage under a heat lamp for 5 min to dilate the tail veins. The 3-month-old mice were anesthetized with isoflurane and intracardially perfused with 40 mL of saline (0.9% NaCl) 1, 2, or 6 h after the injections. 11-month-old mice received single i.v. injections of rh Bri2 BRICHOS R221E monomers or of rh WT Bri2 BRICHOS-AU1,[28] 10 or 20 mg/kg, or an equal volume of PBS. They were anesthetized and perfused 2 h after the injections. Brains were quickly removed, snap frozen in dry ice, and stored at −80°C until analysis.

Blood samples from 3-month-old mice (n = 5) were collected from the tail vein 5, 30, 60, 120, and 360 min after rh Bri2 BRICHOS R221E monomers were injected. The lateral tail vein was punctured using a 27G needle, and 50–100 μL of blood was collected at each time point. Blood samples were coagulated and centrifuged at 4°C for 10 min at 3,000 rpm, and serum was collected and stored at −20°C. Before analysis, the samples were diluted 1:5 in 1× PBS.

Mouse brains were homogenized in 50 mM Tris-HCl (pH 7.4), 150 mM NaCl, 1.0% (v/v) Triton X-100, 0.1% (w/v) SDS, and 10 mM EDTA supplemented with protease inhibitors and centrifuged at 4°C for 30 min at 14,000 rpm, and then the supernatant was collected and stored at −20°C. The protein concentrations were determined by the Bradford method. Serum samples were prepared in denaturing buffer containing 2% SDS, 0.03 M Tris, 5% 2-mercaptoethanol, 10% glycerol, and bromophenol blue and heated for 10 min at 96°C. The sample volume for serum was normalized so that 100 μg total proteins were loaded per well. Samples were separated on 10% SDS-PAGE gel under reducing conditions and blotted onto a nitrocellulose membrane (GE Healthcare). Brain samples were prepared in 5× SDS reducing buffer at a ratio of 1:5 and heated at 97°C for 10 min. The sample volume for brain homogenate was adjusted to give 10.3 μg/μL, and 20–150 μg protein was loaded per well. Samples were separated on 16.5% SDS-PAGE gel and blotted onto a nitrocellulose membrane (GE Healthcare). After blotting, the membranes were heated in PBS for 2 min and blocked overnight at 4°C in 5% milk/PBS for 1 h for serum samples and in Intercept blocking buffer for brain homogenates, followed by overnight incubation at 4°C with an in-house goat anti-Bri2 BRICHOS antibody (1:300) in 5% milk, 0.1% Tween/PBS for serum samples or (1:400) in Intercept blocking buffer for brain samples incubated for 2 h at room temperature (RT). The membranes were washed three times with 0.1%

Tween/PBS and incubated for 1 h at RT with a secondary anti-goat antibody (1:10,000) for serum samples or IRDye 800CW donkey anti-goat immunoglobulin G (IgG) secondary antibody (LI-COR Biosciences, USA) (1:10,000) for brain samples. After washing, images were acquired using a fluorescence imaging system (LI-COR Biosciences, Odyssey CLx), and band intensity was measured with ImageJ software. For detection and quantification of rh WT Bri2 BRICHOS-AU1, see the procedures described by Tambaro et al.[28]

Serum half-lives were calculated after densitometric analysis of the rh Bri2 BRICHOS R221E band intensities with ImageJ. The concentrations were expressed as relative intensities and normalized for each curve to the sample intensity at 5 min. The apparent half-life was obtained using GraphPad Prism by a non-linear one-phase decay analysis.

Bri2 BRICHOS and Aβ double staining

Immunohistochemistry staining for Bri2 BRICHOS and Aβ was performed in 5-μm-thick coronal sections of paraffin-embedded rh Bri2 BRICHOS R221E-treated App^{NL-G-F} and App^{NL-F} mouse brain tissue. The sections were de-paraffinized in xylene and re-hydrated in graded alcohol series from 99% to 70%. Brain sections were pre-treated for antigen retrieval in DIVA Decloaker 1× solution (Biocare Medical) in a pressure cooker (Biocare Medical) at 110°C for 30 min. The slides were cooled down at RT for 30 min, washed with Tris-buffered saline containing 0.05% Tween 20 (TBS-T), and incubated with peroxidase blocking solution (Dako) for 5 min. After washing in TBS-T, brain sections were blocked with Background Punisher (Dako) for 10 min. Subsequently, the slides were incubated with a solution containing the primary antibodies (rabbit anti-Bri2 BRICHOS antibody, 1:100 [Atlas], and mouse 82E1 anti-Aβ antibody, 1:800 [IBL]) diluted in Dako (Agilent Technologies) antibody diluent overnight at 4°C. After washing with TBS-T, the sections were incubated for 30 min at RT with MACH 2 Double Stain 2 secondary cocktail containing polymer alkaline phosphatase (AP)-conjugated goat anti-rabbit antibody and polymer horseradish peroxidase (HRP)-conjugated goat anti-mouse antibody (Biocare Medical). HRP immunoreactivity was detected by permanent green (Biosite), and AP was visualized with permanent red kit (Biosite) solution. The sections were counterstained in hematoxylin Mayer, de-hydrated, cleared in xylene, and mounted with DEPEX mounting medium (Merck). Images were acquired using a Nikon Eclipse E800 light microscope linked to a high-resolution camera using a 20× objective and 40× objective and analyzed on ImageJ software (National Institutes of Health, MD).

Behavior studies

Novel object recognition

The arena was a square plastic box (35 × 35) with 20-cm walls. Each mouse was gently placed in a corner facing the opaque wall and allowed to freely explore for 5 min for habituation. After a gap of 2 h, mice were placed in same open box with two similar sample objects (Lego) placed diagonally near the corners of the box and allow to explore them freely. The next day, one of the objects was replaced with another object (egg timer), and mice were allowed to explore the two objects for 5 min. The behavior of each mouse was video monitored and analyzed using EthoVision XT software (Noldus, Wageningen, the Netherlands). The arena was cleaned with 70% alcohol after each session. The discrimination index was calculated by dividing the time spent exploring the new object over the total time spent exploring both objects.

Y maze

The Y maze apparatus, made of gray plastic, consisted of three compartments (36 × 15 cm) that extended from a center platform (15 × 15 × 15 cm). Each mouse was placed in one arm facing the center of the maze and then allowed to explore freely for 5 min. The apparatus was cleaned with 70% ethanol to remove any odor cues between each session. The alternation frequency was determined by dividing the number of altered entries, i.e., not entering a recently visited arm, by the total number of entries-1.

Thioflavin S staining

Coronal sections of 5-μm thickness at the level of the hippocampus were obtained on Superfrost Plus microscope glass slides from paraffin-embedded brain tissue using a microtome and allowed to dry at 37°C overnight. Sections were deparaffinized by washing in xylene and in decreasing (99–70%) concentrations of ethanol, followed by staining with 1% filtered thioflavin S prepared in distilled water for 1 h in the dark at RT. The sections were washed in 70% and 95% ethanol, followed by washing with distilled water. The sections were then incubated with Hoechst nuclear stain, followed by washing with PBS-T three times and covered with PermaFluor water-soluble mounting medium. The sections (4 sections/mouse) were then visualized with a Nikon Eclipse E800 confocal microscope and imaged (Nikon DS-Qi2 camera) at 2× magnification. Plaques were counted (by a blinded observer) from the total surface of the cortex and hippocampus region.

Tissue preparation and immunofluorescence

Mouse tissue sections on glass slides were deparaffinized by washing in xylene and in decreasing (99%–70%) concentrations of ethanol. For antigen retrieval, slides were pressure boiled in citrate buffer solution (0.1 M citric acid and 0.1 M sodium citrate) at 110°C for 5 min and then washed with tap water, followed by PBS-T 0.05% for 5 min each. Sections were then incubated with TNB blocking buffer (0.1 M Tris-HCl [pH 7.5], 0.15 M NaCl, and 0.5% blocking reagent; PerkinElmer, USA) or NGS (normal goat serum; Vector Laboratories, USA) for 30 min at RT. The brain sections were then incubated with anti-Aβ (82 × 10^1; IBL, USA) 1:2,000 in TNB buffer, anti-GFAP (Agilent Technologies, USA) 1:500 in TNB buffer, or anti-Iba1 (Fujifilm Wako, Japan) 1:250 in NGS at 4°C overnight. Thereafter, sections were incubated with biotinylated anti-mouse or anti-rabbit antibodies (Vector Laboratories, UK) 1:200 in TNB buffer or NGS for 2 h at RT and then incubated with HRP-conjugated streptavidin (PerkinElmer; USA) 1:100 in TNB buffer or NGS for 30 min. For signal amplification, samples were incubated for 10 min in tyramide (PerkinElmer, USA) 1:50 in amplification reagent. Finally, samples were incubated for 15 min with slow agitation with Hoechst solution, 1:3,000, in PBS-T, followed by mounting with PermaFluor aqueous mounting

medium (Thermo Scientific, USA), and dried overnight. Between each incubation step, samples were washed three times in PBS-T for 5 min with slow agitation. The sections (4 per mouse) were then visualized with a Nikon Eclipse E800 confocal microscope and imaged with a Nikon DS-Qi2 camera at 2× and 10× magnification for further analysis using ImageJ software (National Institutes of Health, MD, USA).

Western blotting

Cortex tissue (n = 6/group) was homogenized in RIPA buffer (Thermo Fisher Scientific, USA) with complete protease inhibitor mixture (Sigma-Aldrich, USA). The supernatants obtained were subjected to protein concentration determination using the Bradford protein assay (Bio-Rad, USA). 50 μg protein was then loaded onto 4%–20% precast gels (Bio-Rad, USA) and transferred to a 0.2-μm polyvinylidene fluoride (PVDF) membrane (Amersham Hybond, GE Healthcare, Germany). Membranes were blocked with 5% skim milk in TBS-T (0.05% Tween 20), probed with anti-Aβ1–16 (6E10; BioLegend, USA) 1:1,000, kept overnight at 4°C, and then incubated with anti-mouse IgG HRP conjugated 1:5,000 (GE Healthcare, UK). Membranes were washed with TBS-T three times after each incubation step. For CTF α/β fragments of APP, cortex tissue was homogenized in 4× PIPES buffer (Sigma-Aldrich, USA), followed by ultracentrifugation at 70,000 rpm for 30 min. The pellet obtained was resuspended in PIPES buffer and measured for protein concentration using a Bradford protein assay. 2.5 μg/μL protein was incubated at 37°C for 30 min, followed by chloroform/methanol 2:1 (v/v) and extraction with gentle mixing for 30 min. To the white interface obtained after centrifugation (15,000 rpm, 15 min), chloroform/methanol 2:1 (v/v) was added with gentle mixing for 1 h, followed by centrifugation (15,000 rpm, 15 min). The pellet was dried using SpeedVac for 2 min, resuspended in sample buffer (SDS sample buffer containing 9 M urea), and kept overnight at RT. 40 μg protein was then loaded onto 8%–16% precast gels (Bio-Rad, USA), transferred to a 0.2-μm nitrocellulose membrane, and probed with anti-APP C-terminal antibody (Sigma-Aldrich, USA) 1:5,000 for detection of CTFα/β fragments of APP. Anti-β-actin (Sigma-Aldrich, USA) 1:3,500 was used as a loading control. Protein bands were visualized using Amersham Imager 600 (GE Healthcare), and band intensity was measured with ImageJ software and normalized with a loading control.

Statistical analysis

Statistical comparisons were performed using Prism 8 (GraphPad, CA, USA). Normality was checked using a Shapiro-Wilk test. Data across different time points were analyzed by two-way ANOVA with Bonferroni correction for multiple comparisons. All other data were analyzed by multiple t test or two-tailed Student's t test. In cases where four different immunostained sections from the same mouse were analyzed (Figures 2 and 4–7), individual variabilities were considered, and the total variance was derived using a pooled variance approach. Analysis of these data was also carried out in R v.4.1.1[53] with the added packages lme4,[54] emmeans,[55] and ggplot2.[56] The effect of treatment on the amount of plaque was then analyzed using a linear mixed-effects model with a fixed effect of treatment and random intercept effects of mouse ID and slice number, using the lmer function from the lme4 package. Variability of the estimates was reported as standard error of the mean (SEM), and $p < 0.05$ was considered statistically significant.

SUPPLEMENTAL INFORMATION

Supplemental information can be found online at https://doi.org/10.1016/j.ymthe.2022.08.010.

ACKNOWLEDGMENTS

We thank Laura Vossen Engblom at the Center for Biostatistics and Bioinformatics (CBB) at BioNut, Karolinska Institutet for advice on statistical analyses; Maiara Kolbe Nusskopf for help with immunostaining; and Takaomi Saido and Takashi Saito at the RIKEN Center for Brain Science for providing *App* knockin mice. This study was supported by the Swedish Research Council (2016-01967 and 2020-02434 to J.J.), the Swedish Brain Foundation, CIMED, a KI doctoral training grant, a StratNeuro post-doctoral grant, the Loo and Hans Osterman Foundation, the Geriatric Diseases Foundation at Karolinska Institutet, the Hedlund Foundation, the Magnus Bergvall Foundation, the Swedish Society for Medical Research, Olle Engkvists Stiftelse, the Swedish Alzheimer Foundation, the Ahlen-stiftelsen, Stiftelsen för Gamla Tjänarinnor, the Gun and Bertil Stohne's Foundation, Demensförbundet, the Hållsten Research Foundation, the Torsten Söderberg Foundation, and the Karolinska Institutet Research Foundation.

AUTHOR CONTRIBUTIONS

S.M. performed experiments, analyzed data, and wrote the paper. L.G.-A., S.T., A.A., and G.C. performed experiments and analyzed data. P.N. planned and supervised experiments and analyzed data. J.J. conceptualized the study, planned and supervised experiments, analyzed data, and wrote the paper. All authors commented on the manuscript.

DECLARATION OF INTERESTS

The authors declare no competing interests.

REFERENCES

1. Hipp, M.S., Kasturi, P., and Hartl, F.U. (2019). The proteostasis network and its decline in ageing. Nat. Rev. Mol. Cell Biol. *20*, 421–435.

2. Benson, M.D., Buxbaum, J.N., Eisenberg, D.S., Merlini, G., Saraiva, M.J.M., Sekijima, Y., Sipe, J.D., and Westermark, P. (2018). Amyloid nomenclature 2018: recommendations by the International Society of Amyloidosis (ISA) nomenclature committee. Amyloid. *25*, 215–219.

3. Kim, D., and Tsai, L.H. (2009). Bridging physiology and pathology in AD. Cell *137*, 997–1000.

4. Winblad, B., Amouyel, P., Andrieu, S., Ballard, C., Brayne, C., Brodaty, H., Cedazo-Minguez, A., Dubois, B., Edvardsson, D., Feldman, H., et al. (2016). Defeating Alzheimer's disease and other dementias: a priority for European science and society. Lancet Neurol. *15*, 455–532.

5. Hardy, J.A., and Higgins, G.A. (1992). Alzheimer's disease: the amyloid cascade hypothesis. Science *256*, 184–185.

6. Hardy, J. (2017). The discovery of Alzheimer-causing mutations in the APP gene and the formulation of the "amyloid cascade hypothesis. FEBS J. *284*, 1040–1044.

7. Jonsson, T., Atwal, J.K., Steinberg, S., Snaedal, J., Jonsson, P.V., Bjornsson, S., Stefansson, H., Sulem, P., Gudbjartsson, D., Maloney, J., et al. (2012). A mutation in APP protects against Alzheimer's disease and age-related cognitive decline. Nature *488*, 96–99.

8. Kunkle, B.W., Grenier-Boley, B., Sims, R., Bis, J.C., Damotte, V., Naj, A.C., Boland, A., Vronskaya, M., van der Lee, S.J., Amlie-Wolf, A., et al. (2019). Genetic meta-analysis of diagnosed Alzheimer's disease identifies new risk loci and implicates Abeta, tau, immunity and lipid processing. Nat. Genet. *51*, 414–430.

9. Huang, L.K., Chao, S.P., and Hu, C.J. (2020). Clinical trials of new drugs for Alzheimer disease. J. Biomed. Sci. *27*, 18.

10. Hultqvist, G., Syvänen, S., Fang, X.T., Lannfelt, L., and Sehlin, D. (2017). Bivalent brain shuttle increases antibody uptake by monovalent binding to the transferrin receptor. Theranostics *7*, 308–318.

11. Aschenbrenner, D.S. (2021). Controversial approval of new drug to treat alzheimer's disease. Am. J. Nurs. *121*, 22–23.

12. Balchin, D., Hayer-Hartl, M., and Hartl, F.U. (2020). Recent advances in understanding catalysis of protein folding by molecular chaperones. FEBS Lett. *594*, 2770–2781.

13. Kampinga, H.H., and Bergink, S. (2016). Heat shock proteins as potential targets for protective strategies in neurodegeneration. Lancet Neurol. *15*, 748–759.

14. Sánchez-Pulido, L., Devos, D., and Valencia, A. (2002). BRICHOS: a conserved domain in proteins associated with dementia, respiratory distress and cancer. Trends Biochem. Sci. *27*, 329–332.

15. Hedlund, J., Johansson, J., and Persson, B. (2009). BRICHOS - a superfamily of multidomain proteins with diverse functions. BMC Res. Notes *2*, 180.

16. Nerelius, C., Martin, E., Peng, S., Gustafsson, M., Nordling, K., Weaver, T., and Johansson, J. (2008). Mutations linked to interstitial lung disease can abrogate antiamyloid function of prosurfactant protein C. Biochem. J. *416*, 201–209.

17. Willander, H., Askarieh, G., Landreh, M., Westermark, P., Nordling, K., Keränen, H., Hermansson, E., Hamvas, A., Nogee, L.M., Bergman, T., et al. (2012). High-resolution structure of a BRICHOS domain and its implications for anti-amyloid chaperone activity on lung surfactant protein C. Proc. Natl. Acad. Sci. USA *109*, 2325–2329.

18. Buxbaum, J.N., and Johansson, J. (2017). Transthyretin and BRICHOS: the paradox of amyloidogenic proteins with anti-amyloidogenic activity for Aβ in the central nervous system. Front. Neurosci. *11*, 119.

19. Kurudenkandy, F.R., Zilberter, M., Biverstål, H., Presto, J., Honcharenko, D., Strömberg, R., Johansson, J., Winblad, B., and Fisahn, A. (2014). Amyloid-beta-induced action potential desynchronization and degradation of hippocampal gamma oscillations is prevented by interference with peptide conformation change and aggregation. J. Neurosci. *34*, 11416–11425.

20. Nerelius, C., Gustafsson, M., Nordling, K., Larsson, A., and Johansson, J. (2009). Anti-amyloid activity of the C-terminal domain of proSP-C against amyloid beta-peptide and medin. Biochemistry *48*, 3778–3786.

21. Poska, H., Haslbeck, M., Kurudenkandy, F.R., Hermansson, E., Chen, G., Kostallas, G., Abelein, A., Biverstål, H., Crux, S., Fisahn, A., et al. (2016). Dementia related Bri2 BRICHOS is a versatile molecular chaperone that efficiently inhibits Abeta42 toxicity in Drosophila. Biochem. J. *473*, 3683–3704.

22. Hermansson, E., Schultz, S., Crowther, D., Linse, S., Winblad, B., Westermark, G., Johansson, J., and Presto, J. (2014). The chaperone domain BRICHOS prevents CNS toxicity of amyloid-beta peptide in Drosophila melanogaster. Dis. Model. Mech. *7*, 659–665.

23. Arosio, P., Michaels, T.C.T., Linse, S., Månsson, C., Emanuelsson, C., Presto, J., Johansson, J., Vendruscolo, M., Dobson, C.M., and Knowles, T.P.J. (2016). Kinetic analysis reveals the diversity of microscopic mechanisms through which molecular chaperones suppress amyloid formation. Nat. Commun. *7*, 10948.

24. Cohen, S.I.A., Arosio, P., Presto, J., Kurudenkandy, F.R., Biverstal, H., Dolfe, L., Dunning, C., Yang, X., Frohm, B., Vendruscolo, M., et al. (2015). A molecular chaperone breaks the catalytic cycle that generates toxic Abeta oligomers. Nat. Struct. Mol. Biol. *22*, 207–213.

25. Leppert, A., Tiiman, A., Kronqvist, N., Landreh, M., Abelein, A., Vukojević, V., and Johansson, J. (2021). Smallest secondary nucleation competent abeta aggregates probed by an ATP-independent molecular chaperone domain. Biochemistry *60*, 678–688.

26. Andrade-Talavera, Y., Chen, G., Kurudenkandy, F.R., Johansson, J., and Fisahn, A. (2021). Bri2 BRICHOS chaperone rescues impaired fast-spiking interneuron behavior and neuronal network dynamics in an AD mouse model in vitro. Neurobiol. Dis. *159*, 105514.

27. Galan-Acosta, L., Sierra, C., Leppert, A., Pouliopoulos, A.N., Kwon, N., Noel, R.L., Tambaro, S., Presto, J., Nilsson, P., Konofagou, E.E., and Johansson, J. (2020). Recombinant BRICHOS chaperone domains delivered to mouse brain parenchyma by focused ultrasound and microbubbles are internalized by hippocampal and cortical neurons. Mol. Cell. Neurosci. *105*, 103498.

28. Tambaro, S., Galan-Acosta, L., Leppert, A., Chen, G., Biverstål, H., Presto, J., Nilsson, P., and Johansson, J. (2019). Blood-brain and blood-cerebrospinal fluid passage of BRICHOS domains from two molecular chaperones in mice. J. Biol. Chem. *294*, 2606–2615.

29. Chen, G., Abelein, A., Nilsson, H.E., Leppert, A., Andrade-Talavera, Y., Tambaro, S., Hemmingsson, L., Roshan, F., Landreh, M., Biverstål, H., et al. (2017). Bri2 BRICHOS client specificity and chaperone activity are governed by assembly state. Nat. Commun. *8*, 2081.

30. Chen, G., Andrade-Talavera, Y., Tambaro, S., Leppert, A., Nilsson, H.E., Zhong, X., Landreh, M., Nilsson, P., Hebert, H., Biverstål, H., et al. (2020). Augmentation of Bri2 molecular chaperone activity against amyloid-beta reduces neurotoxicity in mouse hippocampus in vitro. Commun. Biol. *3*, 32.

31. Saito, T., Matsuba, Y., Mihira, N., Takano, J., Nilsson, P., Itohara, S., Iwata, N., and Saido, T.C. (2014). Single App knock-in mouse models of Alzheimer's disease. Nat. Neurosci. *17*, 661–663.

32. Masuda, A., Kobayashi, Y., Kogo, N., Saito, T., Saido, T.C., and Itohara, S. (2016). Cognitive deficits in single App knock-in mouse models. Neurobiol. Learn. Mem. *135*, 73–82.

33. Shah, D., Latif-Hernandez, A., De Strooper, B., Saito, T., Saido, T., Verhoye, M., D'Hooge, R., and Van der Linden, A. (2018). Spatial reversal learning defect coincides with hypersynchronous telencephalic BOLD functional connectivity in APP(NL-F/NL-F) knock-in mice. Sci. Rep. *8*, 6264.

34. Abelein, A., Chen, G., Kitoka, K., Aleksis, R., Oleskovs, F., Sarr, M., Landreh, M., Pahnke, J., Nordling, K., Kronqvist, N., et al. (2020). High-yield production of amyloid-beta peptide enabled by a customized spider silk domain. Sci. Rep. *10*, 235.

35. Franco-Bocanegra, D.K., George, B., Lau, L.C., Holmes, C., Nicoll, J.A.R., and Boche, D. (2019). Microglial motility in Alzheimer's disease and after Abeta42 immunotherapy: a human post-mortem study. Acta Neuropathol. Commun. *7*, 174.

36. Song, W.M., and Colonna, M. (2018). The identity and function of microglia in neurodegeneration. Nat. Immunol. *19*, 1048–1058.

37. Cai, Z., Hussain, M.D., and Yan, L.J. (2014). Microglia, neuroinflammation, and beta-amyloid protein in Alzheimer's disease. Int. J. Neurosci. *124*, 307–321.

38. Olsen, M., Aguilar, X., Sehlin, D., Fang, X.T., Antoni, G., Erlandsson, A., and Syvänen, S. (2018). Astroglial responses to amyloid-beta progression in a mouse model of alzheimer's disease. Mol. Imaging Biol. *20*, 605–614.

39. Osborn, L.M., Kamphuis, W., Wadman, W.J., and Hol, E.M. (2016). Astrogliosis: an integral player in the pathogenesis of Alzheimer's disease. Prog. Neurobiol. *144*, 121–141.

40. Wyss-Coray, T. (2006). Inflammation in Alzheimer disease: driving force, bystander or beneficial response? Nat. Med. *12*, 1005–1015.

41. Chen, W.T., Lu, A., Craessaerts, K., Pavie, B., Sala Frigerio, C., Corthout, N., Qian, X., Laláková, J., Kühnemund, M., Voytyuk, I., et al. (2020). Spatial transcriptomics and in situ sequencing to study alzheimer's disease. Cell *182*, 976–991.e19.

42. Dolfe, L. (2016). BRICHOS Interactions with Amyloid Proteins and Implications for Alzheimer Disease. PhD Thesis.

43. Dodart, J.C., Bales, K.R., Gannon, K.S., Greene, S.J., DeMattos, R.B., Mathis, C., DeLong, C.A., Wu, S., Wu, X., Holtzman, D.M., and Paul, S.M. (2002). Immunization reverses memory deficits without reducing brain Abeta burden in Alzheimer's disease model. Nat. Neurosci. *5*, 452–457.

44. van Dyck, C.H. (2018). Anti-Amyloid-beta monoclonal antibodies for alzheimer's disease: pitfalls and promise. Biol. Psychiatry *83*, 311–319.

45. Linse, S., Scheidt, T., Bernfur, K., Vendruscolo, M., Dobson, C.M., Cohen, S.I.A., Sileikis, E., Lundqvist, M., Qian, F., O'Malley, T., et al. (2020). Kinetic fingerprints

differentiate the mechanisms of action of anti-Abeta antibodies. Nat. Struct. Mol. Biol. *27*, 1125–1133.

46. Iaccarino, H.F., Singer, A.C., Martorell, A.J., Rudenko, A., Gao, F., Gillingham, T.Z., Mathys, H., Seo, J., Kritskiy, O., Abdurrob, F., et al. (2016). Gamma frequency entrainment attenuates amyloid load and modifies microglia. Nature *540*, 230–235.

47. Martorell, A.J., Paulson, A.L., Suk, H.J., Abdurrob, F., Drummond, G.T., Guan, W., Young, J.Z., Kim, D.N.W., Kritskiy, O., Barker, S.J., et al. (2019). Multi-sensory gamma stimulation ameliorates alzheimer's-associated pathology and improves cognition. Cell *177*, 256–271.e22.

48. Ribary, U., Ioannides, A.A., Singh, K.D., Hasson, R., Bolton, J.P., Lado, F., Mogilner, A., and Llinás, R. (1991). Magnetic field tomography of coherent thalamocortical 40-Hz oscillations in humans. Proc. Natl. Acad. Sci. USA *88*, 11037–11041.

49. Arroyo-García, L.E., Isla, A.G., Andrade-Talavera, Y., Balleza-Tapia, H., Loera-Valencia, R., Alvarez-Jimenez, L., Pizzirusso, G., Tambaro, S., Nilsson, P., and Fisahn, A. (2021). Impaired spike-gamma coupling of area CA3 fast-spiking interneurons as the earliest functional impairment in the App(NL-G-F) mouse model of Alzheimer's disease. Mol. Psychiatry *26*, 5557–5567.

50. Mathys, H., Davila-Velderrain, J., Peng, Z., Gao, F., Mohammadi, S., Young, J.Z., Menon, M., He, L., Abdurrob, F., Jiang, X., et al. (2019). Single-cell transcriptomic analysis of Alzheimer's disease. Nature *570*, 332–337.

51. Kronqvist, N., Sarr, M., Lindqvist, A., Nordling, K., Otikovs, M., Venturi, L., Pioselli, B., Purhonen, P., Landreh, M., Biverstål, H., et al. (2017). Efficient protein production inspired by how spiders make silk. Nat. Commun. *8*, 15504.

52. Zhong, X., Kumar, R., Wang, Y., Biverstal, H., Jegerschöld, C., Koeck, P., Johansson, J., Abelein, A., and Chen, G. (2022). Amyloid fibril formation of Arctic amyloid-β 1–42 peptide is efficiently inhibited by the BRICHOS domain. ACS Chem. Biol. *17*, 2201–2211.

53. R_Core_Team, R. (2021). A Language and Environment for Statistical Computing (R Foundation for Statistical Computing).

54. Bates, D., Mächler, M., Bolker, B., and Walker, S. (2015). Fitting linear mixed-effects models using lme4. J. Stat. Softw. *67*, 1–48.

55. Lenth, R. (2020). Emmeans: Estimated Marginal Means, Aka Least-Squares Means.

56. Wickham, H. (2016). ggplot2: Elegant Graphics for Data Analysis (Springer-Verlag).

YMTHE, Volume *31*

Supplemental Information

Intravenous treatment with a molecular chaperone designed against β-amyloid toxicity improves Alzheimer's disease pathology in mouse models

Shaffi Manchanda, Lorena Galan-Acosta, Axel Abelein, Simone Tambaro, Gefei Chen, Per Nilsson, and Jan Johansson

Supplementary material to

Intravenous treatment with a molecular chaperone designed against amyloid-β toxicity improves features of Alzheimer disease pathology in mouse models

Shaffi Manchanda[1,2], Lorena Galan-Acosta[1,2], Axel Abelein[1,2], Simone Tambaro[2], Gefei Chen[1,2], Per Nilsson[2] and Jan Johansson[1,2,*]

[1]Department of Biosciences and Nutrition, Karolinska Institutet, Neo, 141 83 Huddinge, Sweden

[2]Department of Neurobiology, Care Sciences and Society, Division of Neurogeriatrics, Karolinska Institutet, 171 64 Stockholm, Sweden

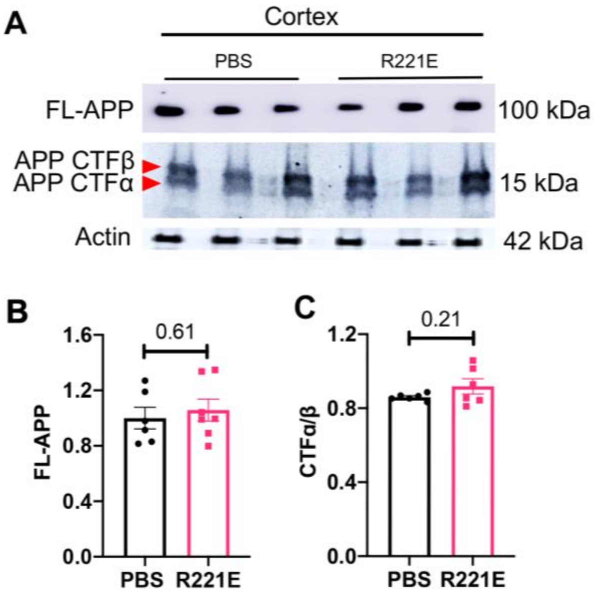

Supplementary Fig. S1. Full-length APP and its processed forms levels remain unchanged after Rh Bri2 BRICHOS R221E treatment in *App*$^{NL\text{-}F}$ mice.

Representative western blots (A) and histograms (B, C) showing levels of full-length APP (FL-APP) and ratio of APP derived C-terminal fragments (CTFα and CTFβ) in cortex of PBS and rh Bri2 BRICHOS R221E treated *App*$^{NL\text{-}F}$ mice (n=6-7 mice/group). Data are represented as Mean ± *SEM*. Unpaired parametric two-tailed t-test was used to calculate the p-values.
